# The Effect of Bariatric Surgery on Menstrual Abnormalities in Saudi Women: A Cross-Sectional Study

**DOI:** 10.7759/cureus.54964

**Published:** 2024-02-26

**Authors:** Lama Alhumaidan, Ghaday M Alrefaei, Abdulrahman M Alfantoukh, Amer S Alsaeri, Mohammed J Almuayrifi, Mohammed Alfehaid, Azzam S Al-Kadi

**Affiliations:** 1 College of Medicine, Unaizah College of Medicine and Medical Sciences, Qassim University, Unaizah, SAU; 2 College of Medicine, King Abdulaziz University Faculty of Medicine, Jeddah, SAU; 3 College of Medicine, Imam Mohammad Ibn Saud University, Riyadh, SAU; 4 College of Medicine, Majmaah University, Al Majma'ah, SAU; 5 Surgery, Taibah University, Madinah, SAU; 6 Surgery, Unaizah College of Medicine and Medical Sciences, Qassim University, Unaizah, SAU

**Keywords:** uterine fibroids, pcos, hormonal imbalance, menstrual abnormalities, bariatric surgery, obesity

## Abstract

Background: Adults in Saudi Arabia are more likely to be obese, which has negative effects on reproductive health, especially for women. While bariatric surgery (BS) provides a sustainable approach, little is known about how it affects menstrual health and requires a study among the Saudi demographic.

Methods: The current investigation is a cross-sectional study conducted in Saudi Arabia. Data were collected using an online questionnaire to assess the impact of BS on menstrual abnormalities in women. Data were cleaned in Excel and analyzed using the Statistical Package for the Social Sciences (IBM SPSS Statistics for Windows, IBM Corp., Version 24.0, Armonk, NY).

Results: This study included 516 Saudi women who underwent various BS procedures, with 37.2% aged 18-30 years and 97.9% residing in Saudi Arabia. Approximately 85.9% underwent sleeve gastrectomy (SG), experiencing a mean weight loss of 54.2 kg. Co-morbidities included polycystic ovary syndrome (PCOS) (12.4%), hypothyroidism or hyperthyroidism (11%), uterine fibroids (4.7%), and hormonal imbalances (2.5%). Post-surgery, 18% encountered BS complications from BS, while 8.3% used antidepressants. Moderate sports participation ranged from 12.2% (five or more days) to 36.2% (one to three days). In particular, no significant associations were found between complications and various parameters, except a marginal association with educational level (p=0.071). The number of menstruations per year did not change statistically significantly, remaining constant at 10.6±4.8 before surgery and 10.6±4.9 after surgery (p = 0.859). However, there was a mild decrease in the amount and duration of menstruation, reported by 23.3% and 27.3% of the participants preoperatively and post-operatively, respectively.

Conclusion: The study demonstrates the impact of BS on menstrual abnormalities in Saudi women. Despite a significant weight reduction, we found mild improvement in the amount and duration of menstruation with no substantial effect observed on the menstrual cycle frequency. Psychological support after surgery is crucial, considering the increased use of antidepressants.

## Introduction

Obesity is a major global health problem, affecting over 650 million adults worldwide and contributing to a wide range of health problems and reduced quality of life [1]. The prevalence of obesity has almost tripled since 1975, making it a significant public health problem [2]. The Kingdom of Saudi Arabia (KSA) is currently facing a severe obesity crisis, with an estimated 35-42% of the adult population affected by this condition [3,4]. In the KSA, a significant challenge is the high prevalence of obesity among women, which has detrimental consequences for their reproductive health [5]. Briggs (2017) found that 32% of women in Saudi Arabia were considered overweight [6]. Women who are obese have a higher chance of experiencing menstrual irregularities and abnormalities due to higher estrogen levels [3]. Menstrual abnormalities, including irregular cycles and dysmenorrhea, are frequently observed in obese women [7]. These abnormalities can cause fertility problems and show an increased risk of developing endometrial hyperplasia and cancer [8]. Furthermore, obesity has been associated with hormonal imbalances, such as polycystic ovary syndrome (PCOS), which can further exacerbate menstrual irregularities and negatively affect reproductive health [9].

Bariatric surgery (BS) is a medical option for weight loss that has gained popularity due to its definitive and efficient approach to treating obesity, according to Rózanska-Waledziak et al. [7]. On a global scale, estimates suggest that 1.3 million bariatric procedures are performed annually [7]. The number of BSs performed in Saudi Arabia has increased substantially over the past decade. The estimated number of BS procedures was between 30,000 and 40,000 per year back in 2020 [7]. BS includes procedures such as Roux-en-Y gastric bypass (RYGB), sleeve gastrectomy (SG), and adjustable gastric banding and has become a successful treatment option for people suffering from obesity [10].

Furthermore, BS leads to significant weight loss and improvements in obesity-related co-morbidities such as type 2 diabetes, hypertension, and sleep apnea [11]. BS has also been shown to improve hormonal imbalances and fertility in women affected by PCOS [12]. BS was also shown to significantly improve the quality of life among morbidly obese patients [4,13]. Many studies have examined the effect of obesity on the mental and physical health of women in Saudi Arabia [14,15]. However, it has rarely focused on the impact of BS on menstrual abnormalities among women in KSA. Alqahtani et al. evaluated the effect of BS on various health parameters in a sample of 500 Saudi patients. This study found that BS was effective in improving obesity-related co-morbidities, including type 2 diabetes, hypertension, and sleep apnea. However, the study did not explicitly evaluate the impact of BS on menstrual abnormalities [16].

As global obesity rates continue to rise, this study aims to learn more about how BS affects menstrual health. Additionally, comparing the results of international, regional, and local studies with the KSA population will help provide a more nuanced understanding of the relationship between BS and menstrual abnormalities in this unique demographic. Therefore, conducting a comprehensive cross-sectional study to assess the impact of BS on menstrual abnormalities in KSA is crucial. In this research, our objective was to analyze the incidence of menstrual abnormalities among women who have undergone BS, estimate the prevalence of hyperandrogenism manifestation and contraception use in women who have undergone BS, and assess overall reproductive health outcomes after BS among women in Saudi Arabia.

## Materials and methods

Study design

The research was carried out using a cross-sectional design in Saudi Arabia using an online questionnaire sent to women who had undergone BS with menstrual abnormalities to assess the impact of surgery on these abnormalities. This study was carried out from April to December 2023. The researchers sought approval from Qassim University, the Faculty of Medicine, and the Research Ethics Committee (CM-REC) (ID No. 23-54-01). Participation was voluntary, and participants were allowed to withdraw their consent to participate in the study.

Sample size

The Raosoft sample size calculator (Raosoft, Inc., Seattle, WA) available at http://www.raosoft.com/samplesize.html was used to calculate the sample size. Keeping the margin of error at 5% and the confidence level at 95%, with the population size of women who had undergone BS, the final recommended sample size was 376. The sample was selected using a convenience sampling technique. The questionnaire was distributed online and included subjects who met the inclusion criteria who had agreed to participate at the time of data collection and excluded subjects who met the exclusion criteria. Nonprobability purpose sampling was employed to select study subjects.

Inclusion and exclusion criteria

The current article involved women living in Saudi Arabia who had undergone BS and had experienced menstrual abnormalities. All men living in Saudi Arabia were excluded from the study. Data were collected from participants who met the inclusion and exclusion criteria. The questionnaire was written by the co-authors, using the following references for some questions [7,17].

Study stools

Participants were asked to provide demographic information, including their residency status in Saudi Arabia, age range, and educational level. Additionally, participants reported details about their BS experience, specifying the type of surgery undergone, the time elapsed since the surgery, pre-surgery weight, current weight, kilograms lost post-surgery, and any complications experienced. They also indicated the frequency of engaging in moderate-intensity physical activities per week. Furthermore, participants were queried about their menstrual history and experiences. This included the age at which they had their first menstrual cycle, current menopausal status, and any irregularities or changes in menstruation patterns pre- and post-operatively. Participants provided information on the frequency and heaviness of menstruation, as well as any signs of perimenopause they may be experiencing. Moreover, they disclosed any diagnoses of conditions such as uterine fibroids, hormonal imbalances, clotting disorders, cancer, sexually transmitted infections, PCOS, or thyroid disorders. Additionally, participants reported their contraceptive use before and after surgery and disclosed any medications they were taking, including heparin, warfarin, antidepressants, corticosteroids, anti-seizure drugs, and levothyroxine. The study measures included demographic information, details regarding BS, and questions related to menstrual history and experiences. These measures aimed to gather comprehensive data to analyze the impact of BS on menstrual abnormalities among women in Saudi Arabia.

Statistical analysis

As a pre-test for the data collection instrument, a pilot study with 10% (51.6) of the participants was conducted to identify any issues and barriers to recruiting participants and evaluate the acceptability of the observation or interview protocol. Data from the pilot study were not included in the Cronbach alpha of the main study, which was used to evaluate the reliability, or internal consistency, of a set of scales or test items in the context of this study. Data were entered, classified, and deleted from missing data before being analyzed with the standard computer program Statistical Package for the Social Sciences (IBM SPSS Statistics for Windows, IBM Corp., Version 24.0, Armonk, NY). To evaluate the association between two or more qualitative variables, the Chi-square test (χ2) was used. When comparing two quantitative normally distributed variables, the student's t-test was used, and the analysis of variance (ANOVA) test was used when comparing more than two quantitative normally distributed variables. A p-value of 0.05 is significant.

Ethical considerations

The researchers sought approval from Qassim University, College of Medicine, Research Ethics Committee (CM-REC) dated September 3rd, 2023. Participation was voluntary, and participants were allowed to withdraw their consent to participate in the study. All data from the survey were kept private, and only researchers could access their information.

## Results

Basic sociodemographic of the study

A cohort of 516 patients with BS is studied to elucidate pertinent demographic and clinical characteristics. It reveals a diverse age distribution, with 37.2% between 18 and 30 years old and 25.2% between 41 and 50 years old. There is a predominant presence of Saudi Arabian residents (97.9%), with 61.6% having a bachelor's or higher degree. Regarding surgical interventions, SG was the procedure performed most frequently (85.9% of cases). A considerable proportion (40.7%) had undergone surgery 1.6-2 years before the study, demonstrating that the study was longitudinal. According to the weight parameters recorded after the operation, the study population lost a significant amount of weight (54.2 kg), demonstrating the efficacy of BS (Table 1).

**Table 1:** Sociodemographic and other parameters of all patients who had bariatric surgery

Sociodemographic parameters		Frequency (n=516)	Percent (%)
Age	< 18 Years	9	1.7
	18-30 Years	192	37.2
	31-40 Years	148	28.7
	41-50 Years	130	25.2
	> 50 Years	37	7.2
Live in the Kingdom of Saudi Arabia	Yes	505	97.9
	No	11	2.1
Education	No Education	5	1.0
	Primary School	7	1.4
	Secondary School	21	4.1
	Higher School	97	18.8
	Diploma	68	13.2
	Bachelors or Higher Education	318	61.6
Type of bariatric surgery	Laparoscopic adjustable Gastric banding	33	6.4
	Roux-En-Y	40	7.8
	Sleeve gastrectomy	443	85.9
How long your surgery has been done	1-6 Months	116	22.5
	7-12 Months	92	17.8
	1-1.5 Years	92	17.8
	1.6-2 Years	210	40.7
Weight parameters (Kg)	Before Surgery (Mean)	130.7 Kg	
	Current Weight (Mean)	76.5 Kg	
	Weight Loss After Surgery (Mean)	54.2 Kg	

Co-morbidity patterns in BS patients

**Figure 1:**
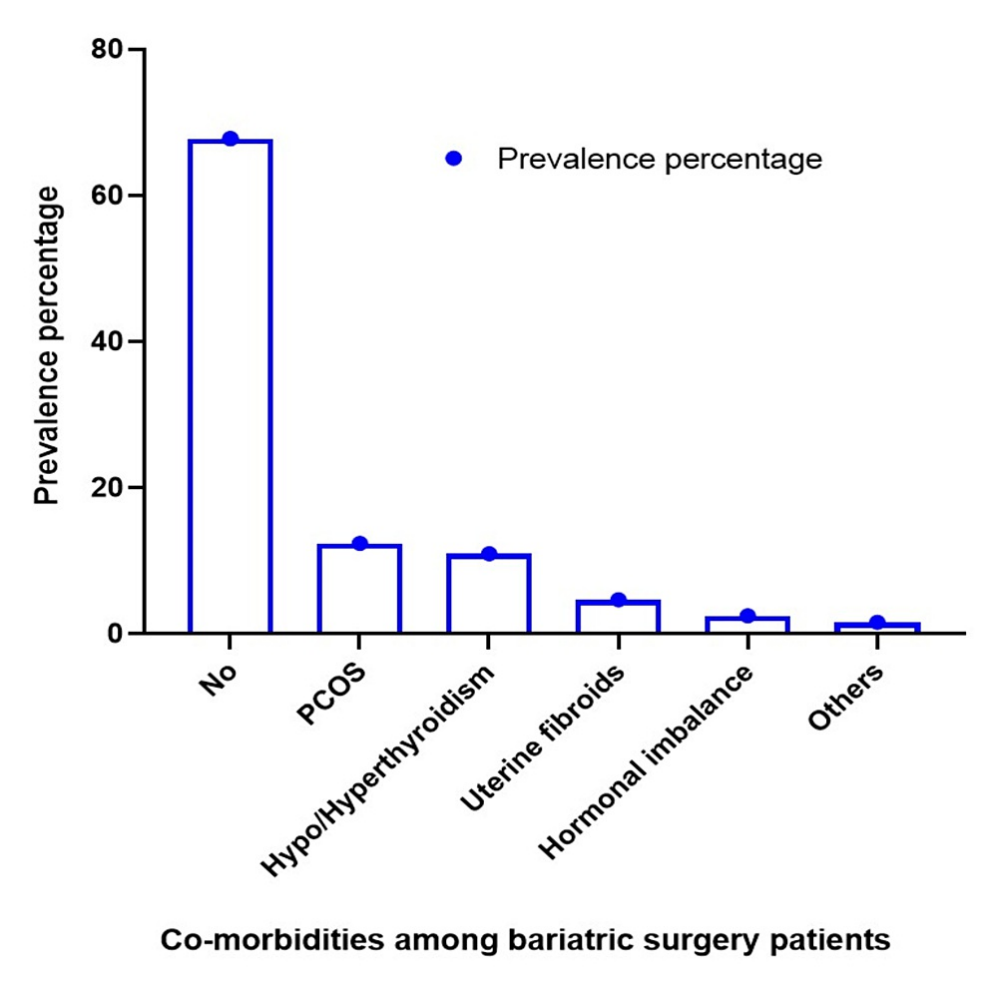
Co-morbidities among bariatric surgery patients PCOS: polycystic ovary syndrome

Based on the results presented in Figure 1, the patients who underwent did not have any comorbidities such as type 2 diabetes mellitus and hypertension (67.8%). The prevalence of PCOS was observed in 12.4% of the patients, indicating an association between obesity and reproductive health issues. There were 11% of the people with hypothyroidism or hyperthyroidism, which emphasizes the endocrine and metabolic interconnection. The incidence of uterine fibroids was about 4.7% among our subjects. However, 2.5% of the patients had hormone imbalances, highlighting the systemic effects of obesity. Among those undergoing BS, a small percentage (1.6%) had unspecified co-morbidities, which warrants further study to understand their health landscape.

BS complications and their varied incidences

The results of a sample of 516 people related to complications after BS are presented here (Table 2). Most respondents, 82.0%, reported no complications after surgery, while 18.0% acknowledged complications, but the majority were minor. Gastric leak was explicitly found in 1% of the respondents, and thromboembolism events occurred in 3.7%. Post-operative bleeding was reported to be about 2.3%. Dumping syndrome and vitamin deficiency accounted for 1.7% of the most frequently reported complications. On the contrary, complications such as hair loss, anemia, gallstones, gastroesophageal reflux disease (GERD), gastric ulcers, gastric obstructions, sagging skin, persistent vomiting, hypoglycemia, loss of libido, menstrual disorders, and mood disorders were found to have lower rates ranging from 0.2% to 1.4%. This detailed breakdown emphasizes the multifaceted nature of complications following BS by providing valuable information on the prevalence of specific adverse events within the studied population.

**Table 2:** Presence of complications after bariatric surgery GERD: Gastroesophageal reflux disease

Presence of complications after bariatric surgery	Frequency (n=516)	Percent	
Any complications after surgery			
No	423	82.0	
Yes	93	18.0	
Different types of complications			
Dumping syndrome	9	1.7	
Vitamin deficiency	9	1.7	
Hair loss	7	1.4	
Anemia	6	1.2	
Gallstone	6	1.2	
GERD	6	1.2	
Gastric leak	5	1.0	
Gastric ulcer	3	0.6	
Gastric obstruction	2	0.4	
Saggy skin	2	0.4	
Persistent vomiting	2	0.4	
Hypoglycemia	1	0.2	
Libido loss	1	0.2	
Menstrual disorders	1	0.2	
Mood disturbance	1	0.2	

Comparison of gynecological symptoms pre-/post-BS

Of the 516 individuals, gynecological symptoms pre-/post-BS were analyzed (Table 3). Regarding the onset of menstruation, 80.6% of respondents started menstruation more than five years before surgery and 11.2% within five years after surgery. Menopause is also experienced by 13.8% of respondents. After surgery, menstrual irregularities were reduced from 41.9% to 36.2%, indicating a positive effect of BS. One of the most notable findings was the increase in the number of days in a woman's menstrual cycle reported by 21.7% of respondents. Menstruation that was prolonged and heavy was reported by 23.3% and 27.3% of the participants, respectively, both pre-operatively and post-operatively. It is interesting to note that 17.2% suffered from symptoms of perimenopause (hot flushes). Menstrual health can be affected in a variety of ways by BS, which highlights the need for comprehensive pre-operative and post-operative care.

**Table 3:** Comparison of symptoms pre-/post-bariatric surgery and surgery complications

Comparison of symptoms pre/post-bariatric surgery and surgery complications		Frequency (n=516)	Percent (%)
Starting of menstruating	< 5 years before operation	24	4.7
	< 5 years after operation	58	11.2
	> 5 years before operation	416	80.6
	> 5 years after operation	18	3.5
Currently in menopause	No	445	86.2
	Yes	71	13.8
Suffer from irregular menstruation before operation	No	300	58.1
	Yes	216	41.9
Suffer from irregular menstruation after operation	No	329	63.8
	Yes	187	36.2
Experience increase no. of days in your menstrual cycle	No	404	78.3
	Yes	112	21.7
Prolonged menstruation post-operative	No	396	76.7
	Yes	120	23.3
Heavy menstruation pre-operative	No	375	72.7
	Yes	141	27.3
Heavy menstruation post-operative	No	375	72.7
	Yes	141	27.3
Any signs of perimenopause (hot flushes)	No	427	82.8
	Yes	89	17.2

Contraceptive practices before and after BS

**Figure 2:**
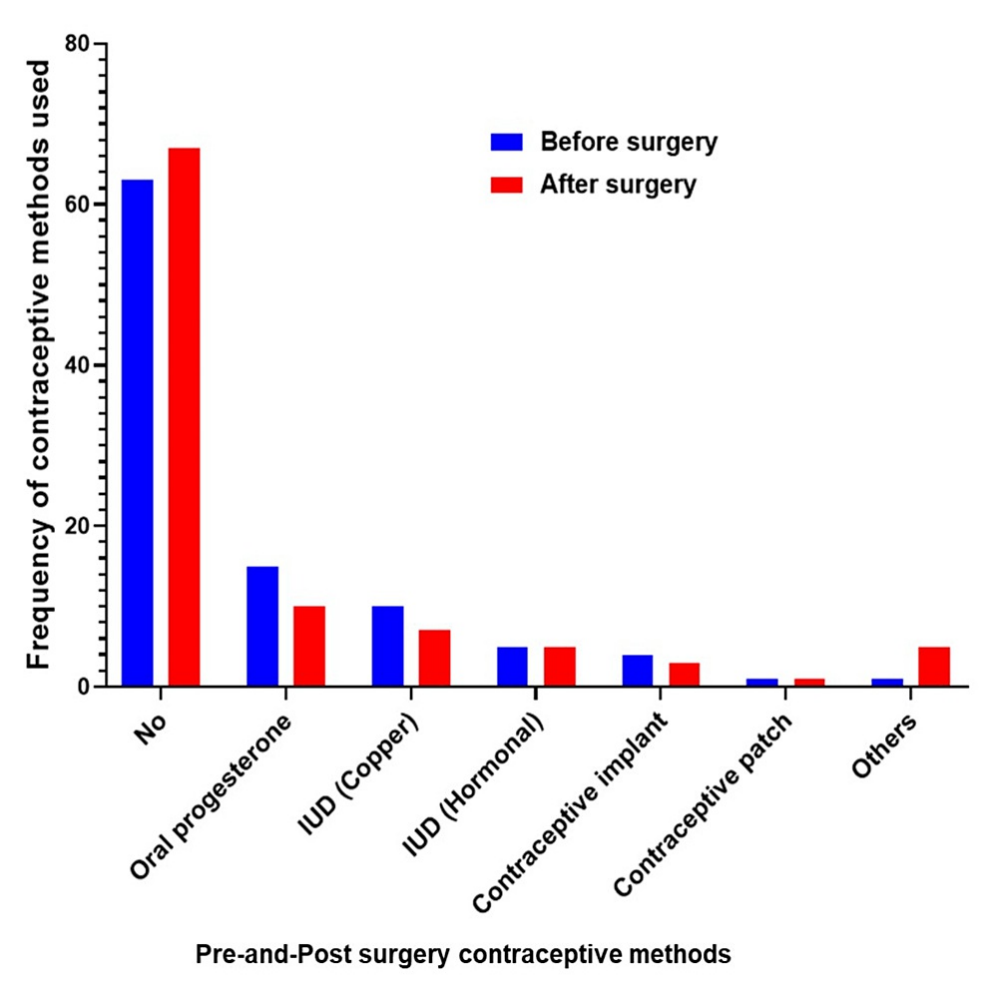
Pre- and post-surgery contraceptive methods IUD: Intrauterine device

Figure 2 illustrates how contraceptive methods are distributed before and after BS. Pre- and post-operatively, 62.8% and 67.6%, respectively, did not use any contraceptive method. A smaller proportion of individuals used oral progesterone, copper intrauterine devices (IUD), and hormonal IUDs before and after surgery. In the population studied, other contraceptives showed a minimal representation.

Physical activity patterns after BS

**Figure 3:**
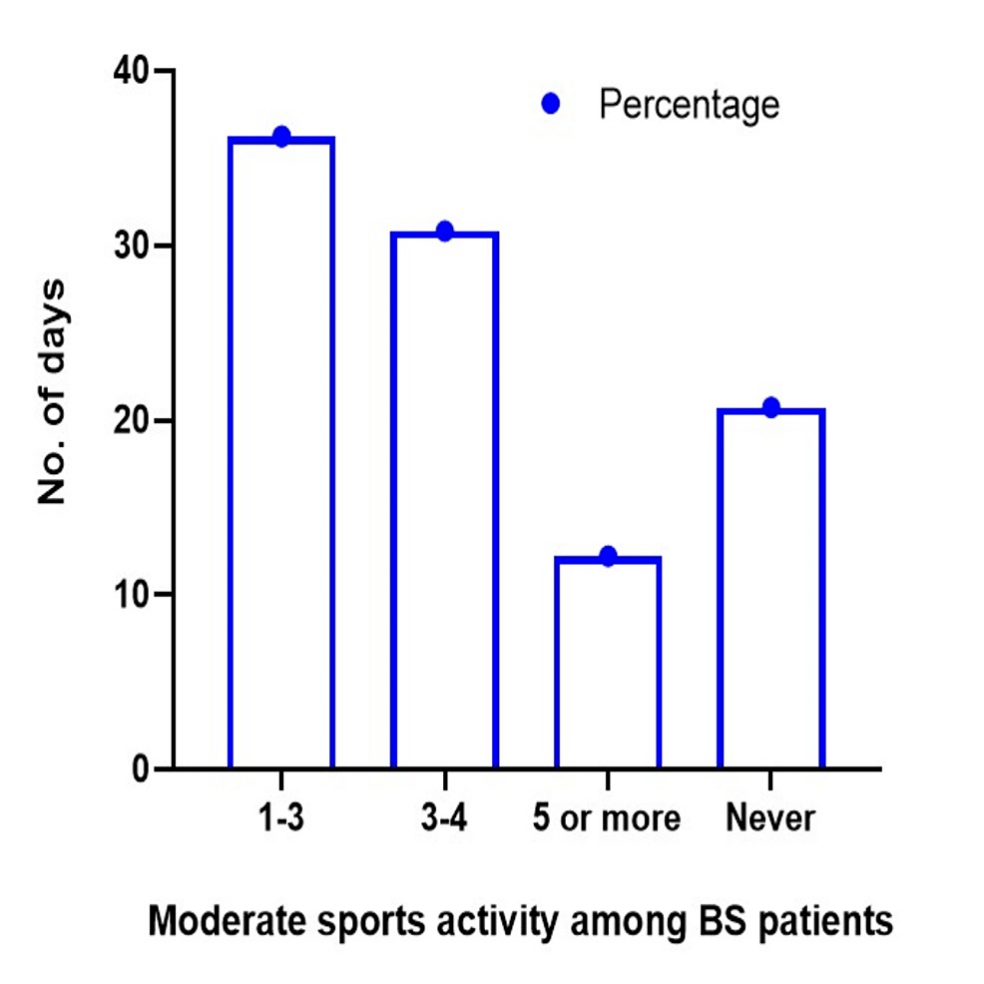
Number of days of moderate sports activity per week among BS patients BS: Bariatric surgery

Figure 3 illustrates the weekly percentage of moderate-intensity sports activities performed by the examined population. A substantial proportion of the respondents participated in sports activities for 1-3 days per week, making up 36.2% of the respondents. Additionally, 30.8% reported participating in sports three or more times a week. On the other hand, 12.2% indicated they participated in sports five or more days a week. The findings show that 20.7% of people do not participate in moderate-intensity sports.

Effect of BS on weight and menstrual cycle

In Table 4, a statistical analysis is performed to determine how BS affects the weight and menstrual cycle parameters within a cohort of 512 individuals. The patients' mean weight decreased from 130.7±433.7 kg before surgery to 76.6±25.4 kg after surgery (p = 0.005). The number of menstruations per year did not change statistically significantly either, remaining constant at 10.6±4.8 before surgery and 10.6±4.9 after surgery (p = 0.859). Despite the substantial and statistically significant impact of BS on weight loss, the findings suggest that the procedure does not significantly influence the frequency of menstruation.

**Table 4:** Effect of bariatric surgery on weight and menstrual cycle (reproductive health)

Effect of bariatric surgery on weight and menstrual cycle		N	Mean ± SD	Sig. Value
Weight (Kg)	Before Surgery	512	130.7 ± 433.7	0.005
	After Surgery	512	76.6 ±25.4	
No. of menstruation (per year)	Before Surgery	487	10.6 ±4.8	0.859
	After Surgery	487	10.6 ±4.9	

Post-operative medication profiles

During this study, 516 patients were analyzed regarding the post-operative pharmacological regimens they adopted after BS (Table 5). All BS patients received anti-coagulations after surgery for 2-3 weeks. However, 3.7% of the participants used anticoagulation medications (heparin and warfarin) were used by 3.7% of participants for six months or less due to thromboembolism events (portal vein or mesenteric veins). About 8.3% of the patients have reported using antidepressants (Selective serotonin reuptake inhibitors (SSRIs)), while the majority (91.7%) do not. Most of the patients abstained from using corticosteroids (prednisone), and only 5.6% used them after surgery. In the same way, only 2.7% of the patients used antiseizure medications (valproic acid and carbamazepine), while 97.3% did not. There was a significant difference between patients who reported taking levothyroxine post-operatively and the majority (85.3%) who did not. The results of this study shed light on the various post-operative medication practices within the studied population and provide information on the pharmaceutical management strategies used after BS.

**Table 5:** Post-operative medications used by patients SSRIs: Selective serotonin reuptake inhibitors

Post-operative medications used by patients		Frequency (n=516)	Percent (%)
Heparin or warfarin	No	497	96.3
	Yes	19	3.7
Anti-depressants (SSRIs)	No	473	91.7
	Yes	43	8.3
Corticosteroid (Prednisone)	No	487	94.4
	Yes	29	5.6
Anti-seizures (Valproic Acid, Carbamazepine)	No	502	97.3
	Yes	14	2.7
Levothyroxine	No	440	85.3
	Yes	76	14.7

Insights into post-operative complications after BS

This study examines post-operative complications related to various sociodemographic and procedural variables among patients with BS (Table 6). Post-operative complications were not statistically significantly associated with age groups (p = 0.761). Furthermore, there was no statistically significant correlation between nationality and post-operative complications in residents of the KSA (p = 0.698).

**Table 6:** Association of post-operative complications with sociodemographic and other parameters

Association of post-operative complications with sociodemographic and other parameters		Post-operative complications		Sig. Value
		No	Yes	
Age	< 18 Years	7	2	0.761
	18-30 Years	162	30	
	31-40 Years	122	26	
	41-50 Years	103	27	
	> 50 Years	29	8	
Nationality of the Kingdom of Saudi Arabia	Yes	413	92	0.698
	No	10	1	
Type of bariatric surgery	Adjustable laparoscopic gastric banding	24	9	0.313
	Roux-En-Y	37	10	
	Sleeve gastrectomy	368	75	
Educational level	No education	3	2	0.071
	Primary school	5	2	
	Secondary school	13	8	
	Higher school	80	17	
	Diploma	54	14	
	Bachelors or higher education	268	50	
Time since surgery	1-1.5 years	76	16	0.516
	1-6 months	99	17	
	1.6-2 years	167	43	
	7-12 months	77	15	
Moderate sports no. of days/week	1-2	156	31	0.491
	3-4	127	32	
	5 or more	55	8	
	Never	85	22	
Starting of menstruation	< 5 years before operation	18	6	0.150
	< 5 years after operation	43	15	
	> 5 years before operation	345	71	
	> 5 years after operation	17	1	

No significant association was observed between the type of BS and complications (p = 0.313). A potential association with educational levels was also observed (p = 0.071), although it did not reach statistical significance. Post-operative complications were not significantly related to the duration of the surgery (p = 0.516). Additionally, there was no significant association between moderate sports activities per week and complications (p = 0.491). No association was found between the beginning age of menstruation and post-operative complications (p = 0.150). These associations contribute valuable information on the complex nature of post-operative complications, emphasizing the need for more research and personalized approaches to post-baby surgery care.

## Discussion

Obesity, a pressing global health problem, mainly affects Saudi Arabian women, leading to potential reproductive health complications. BS, which has become increasingly popular for weight loss, shows efficacy in improving co-morbidities and hormonal imbalances, although its impact on menstrual health remains understudied in KSA. This study presents crucial information on the effects of BS on menstrual abnormalities and various aspects of reproductive health among Saudi women. A comprehensive examination of patient demographics, co-morbidities, post-operative symptoms, contraceptive practices, physical activity, weight changes, medication usage, and their associations offers a holistic understanding of the complex interaction between BS and reproductive health outcomes.

The prevalence of women aged 18-40 years who underwent BS signifies a growing trend to address obesity in younger individuals, which may reflect the importance of early intervention in weight management [18]. The high percentage of participants with a bachelor's degree or higher suggests that education may influence the decision-making process for BS. This suggests the interplay of socioeconomic factors and access to healthcare in this context [19]. SG, which has emerged as the most common surgical procedure, aligns with its effectiveness in achieving substantial weight loss and improving co-morbidities [20,21]. During an SG, 70-95% of the stomach is removed, decreasing the size of the gastric reservoir and accelerating the transit time of nutrients, thereby decreasing nutrient absorption [22]. However, a substantial reduction in average weight of 54.2 kg after surgery underscores the success of bariatric procedures in promoting sustainable weight management and improving general health outcomes.

In particular, there are known and common side effects and complications that occur after BS, such as chronic nausea or vomiting, diarrhea, and fatigue. These findings are consistent with the reported research on similar complaints [23]. Among the factors associated with BS are vomiting, which occurs in 30% of patients after surgery, and a disturbed eating habit that makes supplement compliance challenging. Similarly, many patients with BS have trouble swallowing their medications and/or forget to take supplements [24]. Borbély et al. show that diarrhea is common after bariatric procedures, mainly those with malabsorptive elements such as RYGB and biliopancreatic diversion [25]. A BS could cause intestinal malabsorption of the vitamin B complex due to the involvement of the duodenum, jejunum, and ileum in vitamin absorption [26]. According to Calapkorur and Küçükkatirci [27], the prevalence of vitamin B12 deficiency increased to 61.8% five years after the RYGB operation. An analysis of 75 patients followed for a longer period and without supplementation revealed that 61.8% had low vitamin B12 levels [28]. Malabsorption and insufficient food intake have been reported to be the main reasons for vitamin B12 deficiency in patients who underwent surgery BS [29]. The appearance of less common problems such as hair loss, anemia, and gastric complications corresponds to the previous literature that mentions the diverse spectrum of potential challenges [30]. An assessment of the relationship between BS and diet quality concluded that individuals undergoing gastric banding were more likely than those undergoing SG or RYGB to experience gastrointestinal symptoms and food intolerances during the first year. Furthermore, the SG population had better food intolerance than the RYGB population [31].

Changes in menstrual symptoms and patterns after BS reflect potential impacts on reproductive health. Pg Baharuddin et al. state that BS will improve factors related to ovulation and lead to spontaneous fertility [30]. A notable complication is irregular menstruation, affecting 36.2% of participants post-operatively. This contrasts with the 41.9% who reported such symptoms before surgery. The observed decrease in irregular menstruation after surgery is an intriguing finding that aligns with the potential positive impact of BS on reproductive health [30]. It should be noted that 24.66% of patients with PCOS experienced their first menstruation within seven days after laparoscopic sleeve gastrectomy (LSG). However, this phenomenon is unclear and may be attributed to changes in gastrointestinal hormones, insulin resistance, and the hypothalamic-pituitary-adrenal axis following LSG [32]. In a recent study, 117 patients who underwent SG achieved pregnancy (31%), and no complications were associated with their pregnancies [33]. Although previous literature has mentioned changes in menstrual patterns after BS, the detailed analysis in our study indicates a potential improvement in irregular menstrual cycles [9]. Post-BS, 21.7% experienced an increase in menstrual cycle length, possibly linked to hormonal changes. Prolonged menstruation in 23.3% before BS raises concerns about endometrial health, urging further exploration of its prevalence and underlying mechanisms in this population. It is well known that sex hormones play a crucial role in regulating the menstrual cycle and fertility, which can be disrupted in women who are obese. At both the three and six-month follow-up periods, Anbara [34] found that follicle-stimulating hormone (FSH) and luteinizing hormone (LH) levels increased significantly in the BS group compared to the control group. After BS, several studies have reported improvements in menstrual irregularities, fertility, and hormone profiles [34,35]. However, alterations in reproductive hormones can also contribute to obesity-related fertility problems [36]. The substantial proportion of women who abstain from contraceptive methods after surgery warrants further exploration of the factors that influence this choice. Understanding the reasons for the decrease in contraception use can inform the development of targeted reproductive health counseling and family planning interventions to ensure the promotion of safe and effective contraceptive practices after BS [37].

The prevalence of moderate sports activity among participants highlights the importance of promoting regular physical activity as a vital component of post-operative care. Santos et al. state that physical activity after surgery BS may be associated with additional weight loss and more effective long-term weight control [38]. However, the notable percentage of people who report not participating in moderate sports emphasizes the need for customized interventions to encourage and sustain physical activity after BS. Indirect calorimetry (VO2 max) and walking tests showed that participating in an exercise program after BS improved cardiorespiratory fitness [39]. A study by Bellicha et al. reported that 60% of the participants achieved the aerobic exercise guidelines by participating in moderate to vigorous physical activity (MVPA) for 150 minutes a week, five years after BS. However, only one-fourth managed to attain the preferred duration of 250 minutes of MVPA per week. This is suggested as an efficient method to prevent weight rebound after diet-induced weight reduction [40]. Significant weight loss after bariatric procedures underscores their effectiveness in improving overall health parameters despite a lack of significant impact on the menstrual cycle frequency. However, this weight reduction may have long-term implications for hormonal balance and reproductive health, which warrants further longitudinal investigations. Although the limited use of post-operative medications highlights the success in managing weight-related co-morbidities, the prevalence of antidepressant use emphasizes the need for comprehensive mental health support to address emotional and psychological changes post-surgery [41]. Plaeke et al. found that antidepressant treatment was associated with reduced weight loss after gastric bypass surgery [42].

There are no significant associations between most sociodemographic factors and post-operative complications, suggesting that individual physiological responses to surgical intervention might primarily influence observed outcomes independent of external demographic variables. However, the marginal association between educational level and post-operative complications warrants further investigation into the potential links between education, health literacy, and post-operative health outcomes.

The study has some limitations that should be discussed. First, this study relied on convenience sampling, which may not fully represent population diversity. Second, the study relied on self-reported data, which may be subject to recall bias. Third, the study's cross-sectional design limits the ability to establish causality or assess changes over time. Longitudinal studies are required to provide a more complete understanding of the long-term effects of BS from surgery on menstrual health. Finally, the study was conducted in Saudi Arabia, and the findings may not be directly generalizable to other populations with different cultural, socioeconomic, or healthcare system characteristics. More research is required to validate these findings in various settings.

## Conclusions

This study shed light on the intricate relationship between BS and menstrual abnormalities among Saudi women. Despite a significant weight reduction, we found mild improvement in the amount and duration of menstruation with no substantial effect observed on the menstrual cycle frequency. Psychological support after surgery is crucial, considering the increased use of antidepressants. The findings provide a foundation for further research and the development of customized interventions and personalized care approaches to optimize reproductive health outcomes and overall well-being in this population. Further exploration of the long-term implications of surgery BS on menstrual abnormalities and reproductive health parameters may significantly improve understanding and guide the implementation of comprehensive post-operative care strategies tailored to the needs of this demographic of patients.
